# Emerging Roles of Dystroglycan in Cardiac Remodeling, Fibrosis, and Heart Failure

**DOI:** 10.3390/ijms27177948

**Published:** 2026-09-07

**Authors:** Bhola Shankar Pradhan, Michał Mączewski

**Affiliations:** Department of Clinical Physiology, Centre of Postgraduate Medical Education, 99/103 Marymoncka Str., 01-813 Warsaw, Poland; bhola.pradhan@cmkp.edu.pl

**Keywords:** dystroglycan, heart failure, cardiac remodeling YAP

## Abstract

The dystrophin–glycoprotein complex (DGC) is a structural and signaling network of cardiac muscle. It connects the extracellular matrix to the intracellular cytoskeleton. Dystroglycan, a component of the DGC, plays an essential role in maintaining the integrity of the sarcolemma of cardiac muscle. It contributes to sarcolemma stability, force transmission, mechanotransduction, calcium homeostasis, and cardiomyocyte survival. While the role of dystroglycan signaling is well established in many inherited disorders such as Duchenne muscular dystrophy, it is less studied in acquired heart failure with reduced ejection fraction (HFrEF). Emerging evidence suggests that dystroglycan remodeling may also occur in HFrEF. This review critically analyzes dystroglycan signaling in healthy and failing hearts, with particular emphasis on its emerging role in acquired HFrEF, including cardiac remodeling and fibrosis.

## 1. Introduction

Heart failure with reduced ejection fraction (HFrEF) is an important cause of cardiovascular mortality [1]. In HFrEF, the heart is unable to pump blood sufficiently to meet the metabolic demands of the body or can do so only at the expense of elevated filling pressures [2,3,4]. Due to this, the symptoms associated with HFrEF are dyspnea, fatigue, exercise intolerance, pulmonary congestion, and peripheral edema. The progressive development of HFrEF is initiated by myocardial injury or chronic cardiac stress due to various conditions such as ischemic heart disease, dilated cardiomyopathy, hypertension, myocarditis, valvular heart disease, and inherited cardiomyopathies [5]. This leads to reduced myocardial contractility, leading to decreased stroke volume and reduced left ventricular ejection fraction.

The clinical manifestations of HFrEF are the consequence of progressive myocardial remodeling and fibrosis, electrical dysfunction, and impaired excitation–contraction coupling that reduces ventricular function [6]. However, the mechanisms that regulate these symptoms are barely understood. Recently, disruption of the DGC was associated with myocardial dysfunction, suggesting that the DGC may play a role in the regulation of cardiac function [7,8,9]. The DGC is an important structural component of cardiomyocytes, as it mechanically links the intracellular actin cytoskeleton to the laminin-rich extracellular matrix across the sarcolemma [10]. The DGC is concentrated at the sarcolemma, costameres, and transverse tubules, where it plays a role in stabilizing the cardiomyocyte membrane [10]. This function is important during the repetitive mechanical contraction of the heart, and it also facilitates efficient transmission of contractile force. Dystroglycan is the central transmembrane receptor of the DGC, which binds to laminin and other extracellular matrix proteins outside the cell and anchors dystrophin and the cortical actin cytoskeleton inside the cell [10]. This is essential for maintaining sarcolemmal integrity and preserving the cardiomyocyte architecture during continuous mechanical loading in the heart. Evidence from both human heart failure and experimental cardiomyopathy suggests that remodeling or disruption of Dec components occurs in acquired forms of HFrEF, including ischemic cardiomyopathy, viral myocarditis, and dilated cardiomyopathy [8]. A human failing myocardium exhibits altered expression and organization of dystrophin and dystroglycan, suggesting that destabilization of the DGC is involved during pathological ventricular remodeling. Therefore, there is a need to understand the role of dystroglycan in HFrEF, as alterations in dystroglycan or the DGC are associated with both inherited and acquired cardiomyopathies associated with reduced ejection fraction [8,10].

The force generation by the contractile elements of the myocardium results in continuous physical stress in the heart. There is a requirement for dedicated membrane architecture for the preservation of sarcolemma integrity and the coordination of biomechanical and biochemical signals against this stress [7,11,12]. Located at the sarcolemma and T-tubule regions, the DGC is a highly conservative multicomplex of proteins. It comprises various subunits, such as dystrophin, α- and β-dystroglycan, sarcoglycans, sarcospan, syntrophin, and dystrobrevins, along with signaling components, linking the cytoskeleton (actin) to the extracellular matrix (laminin). It is responsible for proper transfer of forces, defense against contractions, regulation of mechanosignal transduction, calcium (Ca^2+^) buffering, nitric oxide (NO) production, and plasma membrane excitability [7,13,14,15].

Dystrophin has been the principal focus of DGC research, as mutations in the dystrophin (DMD) gene lead to Duchenne and Becker muscular dystrophies, and dystroglycan (DG) is the core connector of the complex. The α-dystroglycan anchors the DGC to extracellular matrix proteins such as laminin, perlecan, and agrin. It further connects to the membrane-spanning β-dystroglycan and then connects dystrophin to downstream intracellular signaling molecules. This network effectively provides a direct physical and biochemical link between the extracellular matrix and the cardiomyocyte cytoskeleton [16,17,18]. Importantly, in addition to this structural capacity, dystroglycan serves as a hub for signaling processes, including calcium entry, nitric oxide signaling, redox homeostasis, Hippo/Yap signaling, integrin pathway activation, and fibrosis and hypertrophy pathways [7,19,20,21].

DGC integrity has been proven crucial for inherited cardiomyopathies. Defects in dystrophin and dystroglycan O-glycosylation result in X-linked dilated cardiomyopathy, Duchenne muscular dystrophy and dystroglycanopathies, often characterized by the development of ventricular dysfunction, fibrosis, and heart failure [11,22,23]. Mechanistically, the integrity of the DGC limits membrane destabilization, increases the threshold for calcium entry, restricts calcium overload during contraction and protects against oxidative stress, mitochondrial dysfunction, extracellular matrix remodeling and fibrofatty substitution. It also stabilizes the signaling domains that recruit ion channels and maintain calcium regulation [10,13,24].

The role of dystroglycan in HFrEF is still unknown. However, the current literature is focused only on the role of dystrophin dysregulation or global DGC disturbances without further examining dystroglycan alone. It has yet to be analyzed whether the expression levels of β-dystroglycan, O-glycosylation status of α-dystroglycan, and DGC-dependent signaling are affected in distinct ways in the course of HFrEF. Additionally, whether dystroglycan contributes to cholinergic receptor organization, electrical remodeling, and ventricular dysfunction in HFrEF has yet to be elucidated. Further study will reclassify dystroglycan not as a structural protein but as a dynamic membrane regulator of the heart, thus providing novel opportunities for the treatment of heart failure. This review will discuss the key questions of dystroglycan biology in acquired heart diseases such as HFrEF.

## 2. Molecular Organization of Dystroglycan

**Figure 1:**
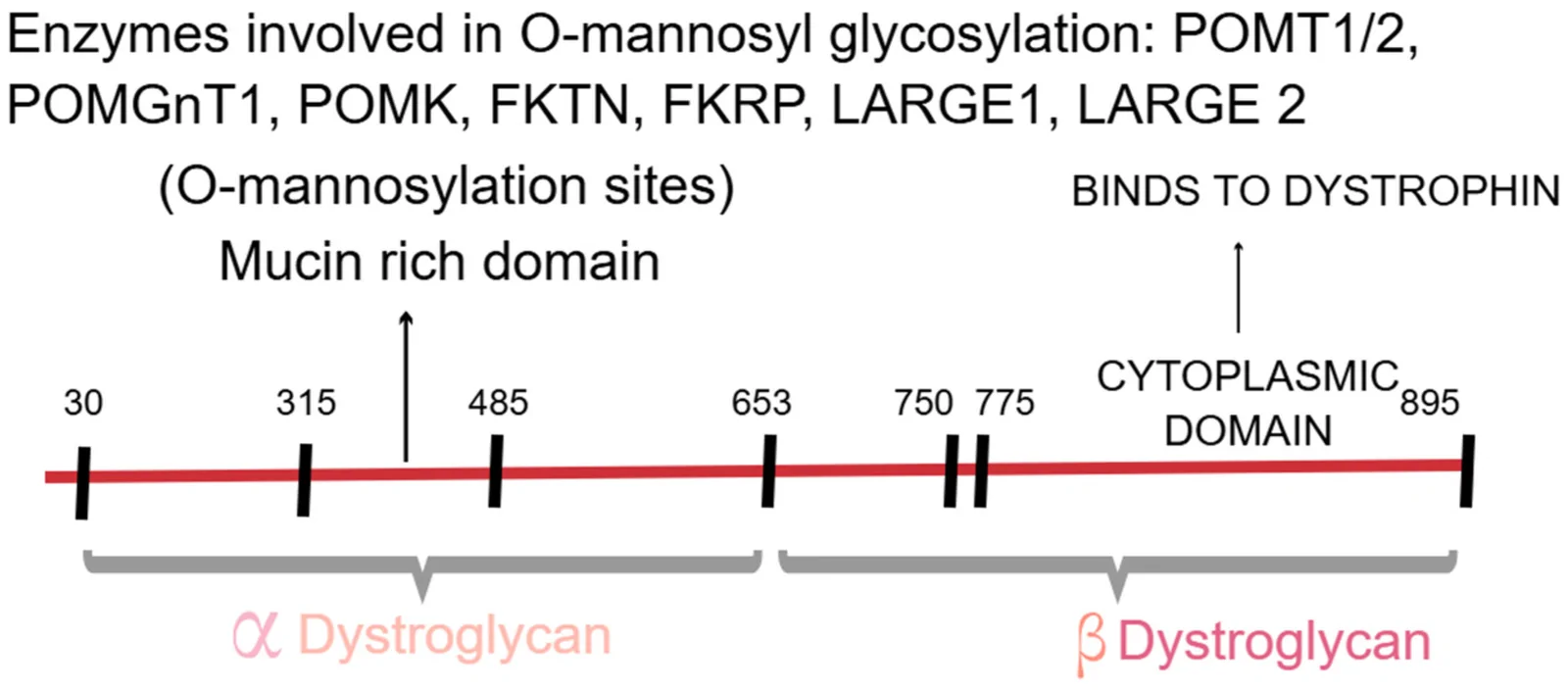
The mucin-rich domain of dystroglycan. The numbers indicate the amino acids in the dystroglycan protein. The red line shows the full length dystroglycan and the black lines denotes the position of amino acid.

Dystroglycan is encoded by the DAG1 gene and expressed as a precursor protein. It further undergoes proteolytic processing, which separates it into a non-transmembrane extracellular α-dystroglycan (α-DG) subunit and an intracellular transmembrane β-dystroglycan (β-DG) subunit. α-DG and β-DG associate with each other in a non-covalent interaction to form the core of the DGC [16,18]. Figure 1 shows the mucin-rich domain of dystroglycan.

The mature α-DG subunit comprises the globular N-terminal domain, a Ser/Thr-rich mu-cin-like central domain, and the C-terminal domain that docks to β-DG [25]. While the terminal domains of α-DG undergo glycosylation with N-linked glycans, the mucin-like domain is subject to extensive modification with O-mannosyl glycans, which are crucial for receptor functionality [26,27]. Key sites for glycosylation, including the mucin core, were identified in amino acids Thr317, Thr319 and Thr379 (depending on the model context) [17,25]. Matriglycan, a repeating xylose–glucuronic acid polymer, is synthesized through serial O-mannosylation (by POMT1/POMT2), modification of a phosphorylated Core M3 glycan and polymerization (by LARGE1/LARGE2). It binds to laminin G-domain-containing ECM proteins with high affinity; these include laminin, agrin, perlecan, pikachurin, thrombospondin [16,25,27,28,29].

Although impaired α-DG glycosylation is the hallmark of dystroglycanopathies, the impact on acquired cardiovascular disease pathologies has been poorly characterized. Specifically, the effects on matriglycan expression levels, site-specific glycosylation profiles and glycan chain lengths under ischemic injury, pressure overload, diabetic cardiomyopathy, or HFrEF are not established. The effect of α-DG on membrane stability, reduced ECM signaling, and pathological cardiac remodeling is unknown.

**Figure 2:**
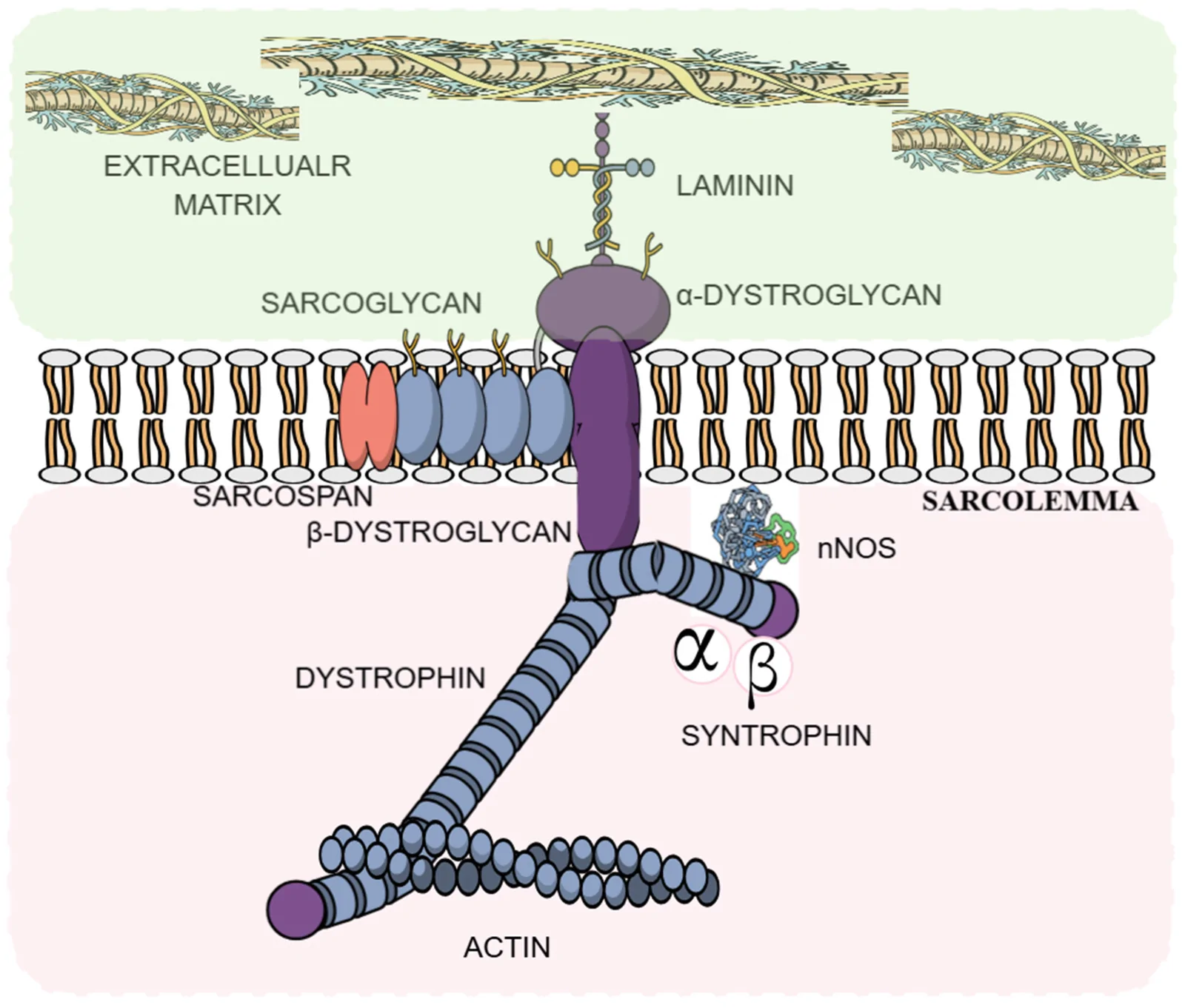
The organization of the DGC complex in cardiomyocytes.

As a single-pass transmembrane protein, β-DG is responsible for tethering α-DG to the sarcolemma and transmitting forces resulting from ECM interactions to the cytoskeleton of the cell [16,30]. The structure of β-DG includes an extracellular N-terminus, a transmembrane region and a well-conserved C-terminal cytoplasmic tail, which binds to dystrophin, utrophin, dystrobrevins, syntrophin, DRP2 and various signaling molecules [12,31,32,33]. Unlike α-DG, the functional role of β-DG is mainly modulated via posttranslational modification; the regulation of the activity of the β-DG protein is especially dependent on tyrosine phosphorylation [30,31,34]. The latter modulates sarcolemmal targeting and incorporation into the DGC, protein trafficking, and downstream signaling molecule recruitment. However, it is not yet understood whether variation in β-DG expression, phosphorylation status and interaction with proteins can be involved in phototransduction, ion channel organization, electrical remodeling and the pathogenesis of HFrEF. Figure 2 shows the organization of the DGC complex in cardiomyocytes.

Dystroglycan is associated with the sarcoglycan–sarcospan (SG–SSPN) complex [12,35,36]. The DGC links the ECM to the cytoskeleton via dystroglycan and stabilizes the DGC against repetitive mechanical stress with the help of the SG–SSPN complex. Loss of any SG leads to instability of the whole complex, resulting in a secondary loss of dystroglycan, dystrophin, and other associated proteins, rendering the membrane fragile and causing cardiomyopathy accompanied by ventricular arrhythmias [35,37,38,39,40]. Sarcospan increased plasma membrane localization of dystroglycan and utrophin and facilitated signaling through syntrophin, dystrobrevins, and neuronal nitric oxide synthase [35,41,42]. Given the contribution of the SG–SSPN complex to the integrity of the DGC, its involvement in acquired cardiac remodeling and HFrEF is unclear.

## 3. Dystroglycan Glycosylation and Functional Importance

One hallmark of α-dystroglycan (α-DG) is extensive mannosyl glycosylation, which is required for high-affinity interactions with laminin and other ECM proteins [25,43]. This interaction is mediated by matriglycan, a unique glycan required to link the DGC to the ECM to maintain sarcolemmal stability and force transmission to the ECM [25,29]. Defects in glycosylation lead to reduced association of the DGC with the ECM [44]. This leads to impaired membrane integrity and the development of alpha-dystroglycanopathies [44]. Alpha-dystroglycanopathies are a class of inherited diseases that may often manifest as dilated cardiomyopathy (DCM), systolic dysfunction, and the eventual progression to heart failure with reduced ejection fraction (HFrEF) [29,43,44,45,46].

Synthesis of matriglycan requires the coordinated action of several glycosyltransferases. Following O-mannosylation initiation by POMT1 and POMT2, a phosphorylated Core M3 glycan is assembled by, for example, POMGNT1, POMGNT2, and B3GALNT2. Ribitol phosphate is added by, for instance, fukutin (FKTN) and fukutin-related protein (FKRP) to create the linkage for elongation. LARGE1 and LARGE2 then polymerize, reiterating xylose–glucuronic acid disaccharides to complete matriglycan synthesis [25,27,28,33,44,47,48].

Mutations in different enzymes along this pathway result in a wide variety of dystroglycanopathies with differing cardiac phenotypes. Defects in POMT1, POMT2, POMGNT1, POMGNT2, and B3GALNT2, which interfere with the production of Core M3 and/or maturation, are related to congenital muscle dystrophies with depressed contractility (systolic dysfunction) and heart failure [44,48]. KRP and FKTN mutations have been strongly linked with progressive cardiomyopathy, ventricular dysfunction and conduction disturbances, including the development of HFrEF despite some affected patients having mild skeletal muscle myopathy [47,49]. In addition, LARGE1 deficiency reduces laminin interactions and destabilizes the DGC, while overexpression of LARGE1 in models of dystroglycanopathy restored matriglycan synthesis and ECM adhesion [25,28,44]. Mutations of dolichol-phosphate mannose synthesis genes, including GMPPB, DPM1–3, DOLK and MPDU1, affect the glycosylation of α-DG and predispose to the onset of ventricular dysfunction and HFrEF [44,48].

Besides the inherited pathologies, deficient α-DG glycosylation leads to impairment of force transmission in the muscle, DGC instability and, thus, enhanced vulnerability to mechanical damage caused by contraction-induced membrane rupture. Even minimal changes in matriglycan expression leads to impairment of transverse tubule formation, disordered calcium homeostasis and elevated mechanical fragility in many studies [16,25,50].

Although there is convincing evidence for inherited dystroglycanopathies, the precise significance of α-DG hypoglycosylation in acquired heart failure is not well defined. Much of our understanding of mechanisms originates from studies of skeletal muscle and animal models, but not from human myocardium. So, it is yet unclear how this aberrant glycosylation impacts DGC–ECM binding, mechanotransduction, calcium handling, fibrosis and ventricular remodeling in HFrEF [25,49,51]. Is the amount of matriglycan and/or glycan chain length modulated in HFrEF, during pressure overload, ischemic heart injury or diabetic cardiomyopathy? What role do compensatory pathways such as LARGE/LARGE2 play in HFrEF [23,52]? Despite encouraging preclinical results with glycosylation-restoring methods, such as overexpressing LARGE1 or supplying ribitol, whether these interventions are effective when targeted to the heart is unknown. It is also not clear whether they have an impact on ventricular remodeling, and HFrEF outcomes have yet to be evaluated in clinical settings or prospective trials [23,28,44]. Also, there is a need to study whether modified α-DG glycosylation is a novel pathogenesis in acquired heart failure.

## 4. Dystroglycan and Sarcolemmal Stability

Each contraction exerts high mechanical stress on the sarcolemma and thus requires prompt repair to protect cardiomyocyte vitality. Dystroglycan, the core component of the DGC protein family, serves as the major functional bridge for transducing mechanical signals and transmitting mechanical force between the ECM and intracellular cytoskeleton. Through its connection with ECM laminin, DGC proteins and dystrophin, dystroglycan efficiently spreads muscle contractility force, maintains the costamere structure, reduces local membrane tension, and defends cardiomyocytes against injuries induced by contraction [53,54].

Another critical factor in sarcolemmal integrity is rapid membrane repair following injury. Upon loss of membrane integrity and calcium influx, membrane repair is driven by the recruitment of repair proteins, such as dysferlin, Mitsugumin 53 (MG53), annexins, and caveolin-3, which work cooperatively to mediate vesicle trafficking, membrane repair, and wound closure [55,56,57]. While both DGC and membrane repair machinery have been well studied, the functional interactions between these two machineries remain undefined. It remains unclear if dystroglycan can directly modulate recruitment/activation of caveolin-mediated repair complexes or if they operate independently of one another [58].

Experiments with cardiac-specific deletion of DAG1 show that dystroglycan is required for the structural integrity of the heart in response to mechanical stress. This genetic deletion leads to the interruption of the ECM–cytoskeletal association, sarcolemmal disintegration, myocardial degeneration, rupture of the sarcolemma, and exercise-induced cardiomyopathy [46]. Interestingly, dystroglycan–agrin interactions control the DGC–Hippo–YAP signaling pathway and also hint that dystroglycan is not just a stabilizer of the membrane; it also controls signals related to regeneration [21,59].

It is crucial to note that the membrane damage in a dystroglycan-deficient heart affects more than a single cardiomyocyte, implying that dystroglycan maintains myocardial integrity at the tissue level. The disruption of DGC compromises the integrity of costameres and intercalated disks, which are essential for mechanical and electrical coupling between neighboring cells [37,50,60,61]. Therefore, calcium overload, oxidative stress, and inflammatory factors can propagate via remodeling gap junctions, mainly connexin43, and exacerbate myocardial damage and electrical instability [62,63,64,65]. Further inflammation, extracellular matrix remodeling and fibrosis are induced through crosstalk with cardiac fibroblasts, endothelial cells and resident macrophages, ultimately leading to maladaptive ventricular remodeling [66,67,68].

The results, therefore, suggest a general protective function of dystroglycan for multicellular integrity in the heart [50]. This concept is important for HFrEF because the sustained low-grade damage to the cell membrane may act as the catalyst for ventricular remodeling. Disrupted membranes lead to an accumulation of calcium, oxygen free radical damage, activation of the inflammasome system, fibroblast activation, extracellular matrix remodeling, and fibrosis. This results in forming a self-sustaining cycle of membrane fragility and progressive systolic function impairment [62,64,69,70,71]. It has not yet been determined whether chronic non-fatal membrane injury precedes cardiomyocyte death or occurs secondary to full-blown heart failure. Also, it is not yet clear whether loss of α-dystroglycan glycosylation alone may contribute to membrane repair deficit. Moreover, there is a need to study whether concomitant therapy of Dec stabilization and membrane repair therapeutic agents, such as recombinant MG53 or annexin A6, may be more beneficial than targeting alone or in combination [72,73,74,75,76].

## 5. Dystroglycan in T-Tubule Organization and Excitation–Contraction Coupling

T-tubules are extensions of the sarcolemma that penetrate deep into the heart muscle cell to allow rapid and uniform spread of electrical charge across the cell. They coordinate with L-type calcium channels and ryanodine receptors (RyRs) for normal cardiac contraction. It is clear from animal and human studies that cardiac contractility depends on healthy T-tubule function.

The T-tubule membrane contains particularly high levels of dystroglycan. Its interaction with the ECM and actin cytoskeleton strengthens the membrane. It prevents disruption caused by mechanical load with repeated excitation–contraction cycles. Moreover, it helps to maintain the specific structural arrangement necessary for efficient excitation–contraction cycles [29,77]. Since laminin binding is reliant on functional α-DG glycosylation, deficiencies in glycosylation machinery, such as FKRP, POMT1, POMT2, POMGNT1, and LARGE, can lead to compromised DGC-ECM interactions. Thus, it leads to a reduction in T-tubule integrity and stability [26,41,47].

Also, DAG1 mutant Zebrafish exhibit gross T-tubule disruption and membrane instability, which underscores the critical role of ECM attachment via dystroglycan [78]. These structural disturbances perturb the distribution of Ca^2+^-handling proteins, including L-type calcium channels, ryanodine receptors, SERCA, and calsequestrin, leading to Ca^2+^ release, reduced Ca^2+^ uptake, Ca^2+^ overload and diminished contractile force output [36,41,77]. To further confirm the importance of this process, a recent study by the Hord group suggests that defects in the glycosylation of α-DG can lead to a progressive accumulation of T-tubule disorganization and abnormal Ca^2+^ handling [29].

T-tubule remodeling in HFrEF leads to the impairment of excitation–contraction coupling, systolic dysfunction, and the progressive deterioration of the heart [7,79]. But whether dysfunction of dystroglycan is an early phenomenon or a secondary effect of heart failure is not fully determined. In Zebrafish and mouse models, it has been shown that dystroglycan dysfunction can lead to severe structural changes in the sarcolemma and impairment of calcium transients and T-tubules, even without major sarcolemmal damage, although most evidence has been derived from inherited dystroglycanopathies [78,80]. In acquired HFrEF, T-tubule remodeling is also caused by oxidative stress, extracellular matrix remodeling, inflammation and microtubule remodeling, which masks the specific contribution of dystroglycan [36,79,81].

It will be interesting to study whether treatment that can restore dystroglycan function actually reverses structural and functional changes associated with T-tubules in failed adult hearts. While attempts to increase glycosylation of α-DG and facilitate DGC–ECM contacts have met some success in developmental and dystroglycanopathy settings, the utility of these approaches for regenerating T-tubules and improving ventricular performance during established HFrEF remains to be addressed. It is also not yet understood if dystroglycan-targeted therapeutics can stand alone or may need to be combined with therapy to affect compensatory regulators of Ca^2+^ handling and microtubule machinery [36,81,82].

## 6. Dystroglycan as a Signaling Platform

In addition to its role in the structural integrity of the DGC complex, dystroglycan serves as a signaling hub that translates information about ECM cues to the intracellular machinery that controls phototransduction. Indeed, the cytoplasmic tail of β-dystroglycan can couple with various kinases, scaffolding proteins, NO synthase-associated signaling pathways, and constituents of the Hippo pathway in such a way that it can enable the muscle to respond appropriately to various mechanical stresses [7,20,21].

One of the well-studied signaling functions of β-dystroglycan is its regulation of the Hippo–YAP pathway that controls heart size and the proliferation and regeneration of cardiomyocytes [20,21]. In postnatal hearts, β-dystroglycan interacts with phosphorylated YAP to restrict its nuclear accumulation, suppressing transcriptional enhanced associate domain (TEAD)-mediated transcription of genes that drive cell proliferation and survival. This mechanism explains why regenerative capacity is lost in the neonatal heart and ties the role of the ECM structure to growth regulation.

Hippo activation leads to YAP phosphorylation and membrane sequestration. In contrast, the binding of agrin to α-dystroglycan releases YAP, which then activates many genes. These findings partly account for the higher regenerative capabilities of neo-nate hearts compared to mature hearts [21,83]. However, cardiac regeneration also depends on other factors such as vascular remodeling, immune responses, ECM remodeling, and signaling through Wnt/β-catenin, extracellular signal-regulated kinase (ERK), and insulin-like growth factor (IGF) pathways, indicating that the dystroglycan–Hippo–YAP axis functions within a broader regenerative network [83,84].

Apart from Hippo–YAP regulation, dystroglycan acts as a transduction platform between ECM sensing and cytoskeletal force transmission, calcium handling and diverse intracellular signals. Dystroglycan anchors the laminin and agrin-rich ECM to the actin cytoskeleton, transducing mechanical stresses into biochemical cues to maintain membrane stability and adaptive responses [7,85,86]. Dystroglycan also cooperates with integrins, focal adhesions, and chemosensitive ion channels such as Piezo proteins to detect ECM mechanics and mechanical strain to regulate nitric oxide signaling, Hippo–YAP activity, and adaptive remodeling [85,87,88].

**Figure 3:**
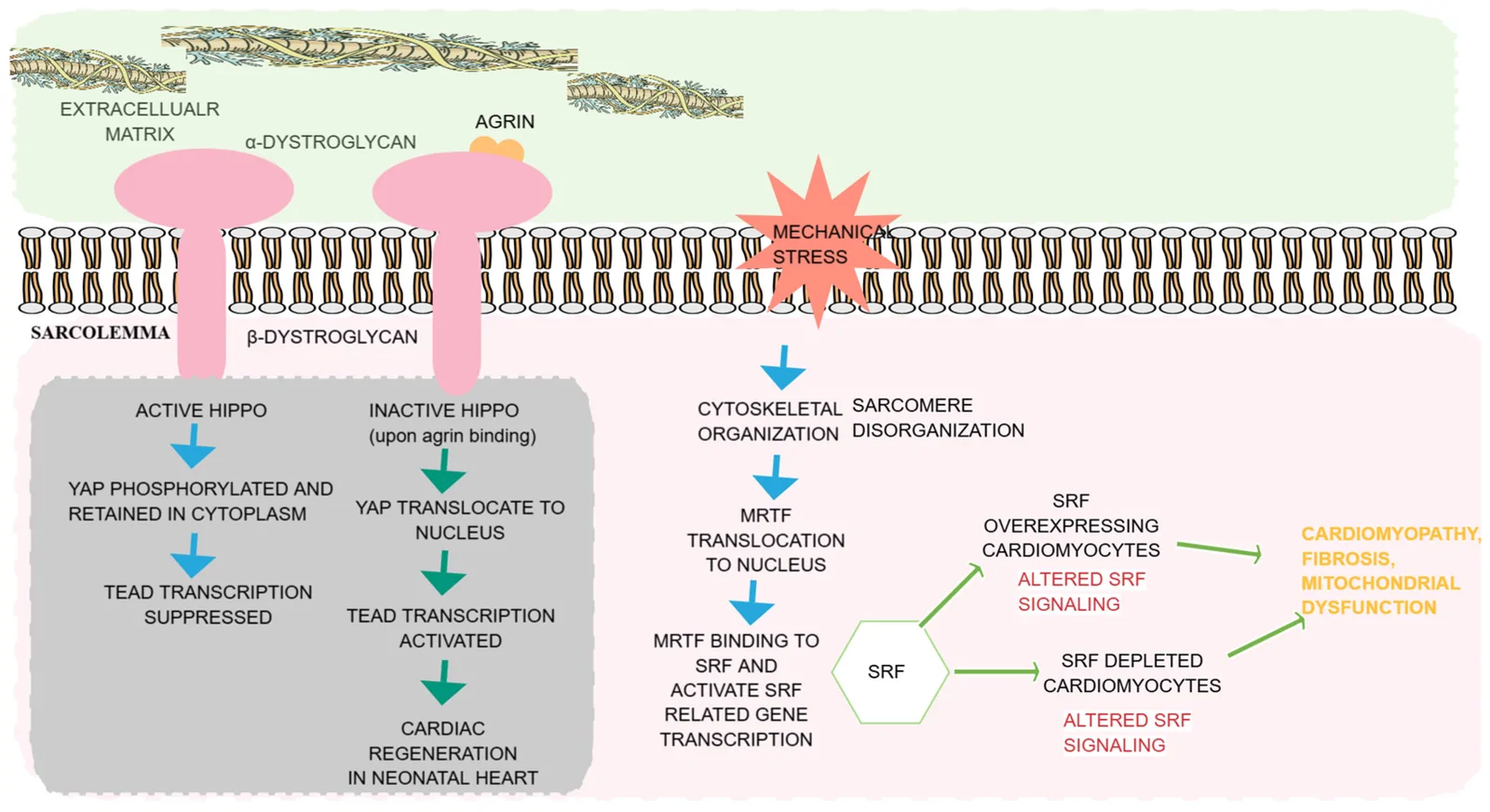
Proposed mechanotransduction model linking dystroglycan, Hippo–YAP, and MRTF–SRF signaling in cardiomyocytes.

Serum response factor (SRF) may act as a transcriptional link between cytoskeletal organization, dystroglycan, and cardiac remodeling. SRF activity is regulated by myocardin-related transcription factors (MRTFs). It responds to changes in actin dynamics and mechanical signals. For example, in cardiomyocytes, sarcomere organization regulates MRTF–SRF signaling. In these cells, ACTN2 promotes F-actin assembly into sarcomeres and decreases G-actin levels, which allows MRTF to enter the nucleus and stimulate transcription through SRF [89]. Cardiac-specific SRF overexpression is associated with altered mitochondrial structure and function, impaired oxidative phosphorylation, increased oxidative stress, abnormal calcium handling, and cardiac fibrosis [90]. Cardiac-specific loss of SRF causes focal myocardial lesions and progressive heart failure [91]. In this model, the authors showed reduced staining for dystrophin due to loss of SRF. However, whether this involves any change in dystroglycan or secondary changes in DEC organization remains unclear. It is interesting to note that dystroglycan binds to F-actin indirectly through dystrophin or directly (as shown in skeletal muscle cells) [92]. This observation suggests that SRF signaling and dystroglycan/DGC organization may be functionally connected in cardiomyocytes. All these suggest that SRF may link changes in sarcomere and dystroglycan/DGC organization with mitochondrial dysfunction and cardiac remodeling. SRF has also been shown to bind the muscle-specific dystrophin promoter, providing a direct transcriptional link between SRF and another component of the DGC [93]. Therefore, it is imperative to assume that changes in dystroglycan/DGC-mediated mechanical signaling may interact with MRTF–SRF-dependent transcriptional responses. Figure 3 shows Hippo and SRF in cardiac cells.

Post-translational modifications also control β-dystroglycan signaling through phosphorylation, proteolytic cleavage, and endocytic internalization [41,49]. Combined with agrin-induced DGC remodeling, these processes control the dynamically regulated level and localization of nuclear YAP. Furthermore, YAP is regulated by ECM microenvironment composition, α-dystroglycan glycosylation, and Hippo activity, as well as interactions with other pathways, such as the ERK, Wnt/β-catenin, IGF, and TGF-β pathways [83,84,94]. While these findings provide strong support for its function as a signaling nexus, it is not known if β-dystroglycan signaling differs between acute (transient activation can mediate repair) and chronic (continued turnover can lead to irreversible remodeling) cardiac disease.

## 7. Dystroglycan Alterations in Inherited Cardiomyopathy

In inherited cardiomyopathies such as DMD, disruption of DGC integrity compromises membrane stability, mechanotransduction, calcium homeostasis, and intracellular signaling, leading to myocardial remodeling, fibrosis, arrhythmias, and heart failure. DMD, caused by mutations and loss of dystrophin, destabilizes the whole complex. Apart from that, mistargeting of β-dystroglycan, α-dystroglycan, sarcoglycans, dystrobrevins, and syntrophin occurs in such cardiomyopathies [10,95,96,97].

Such destabilization inhibits mechanotransduction, nNOS localization, E-C coupling, and Ca^2+^ homeostasis, including ROS production [95,98,99,100]. In parallel, NOS dysfunction, the failure of mitophagy, and the release of mitochondrial damage-associated molecular patterns trigger and exacerbate inflammation, cardiomyocyte death, fibrosis, and ventricle dysfunction [96,98,101]. Hence, DMD cardiomyopathy results from an interconnected cascade of calcium mismanagement, mitochondrial dysfunction, oxidation stress, inflammation, and fibrosis. This favors therapeutic strategies that are able not only to reinforce the cell membrane but also to improve calcium handling and mitochondrial functionality, reduce oxidation stress, and reduce inflammation [97,98,102]. It is still unknown whether the precise correlation between reduced levels of residual α-dystroglycan glycosylation and cardiac disease progression leads to an incomplete genotype–phenotype correlation. Alterations in mechanotransduction, Ca^2+^, NO, mitochondria, Hippo–YAP signaling, and extracellular matrix remodeling due to impaired glycosylation need further study.

On the contrary, in Becker Muscular Dystrophy (BMD), a BMD patient’s dystrophin is partially functional, which slows down disease progression. Although the partial restoration of mechanical signal transduction, NO signaling and Ca^2+^ regulation is responsible for chronic myocardial stress, dysfunction of mitochondria, reactive oxygen species (ROS) formation, inflammation, and progression of dilated cardiomyopathy, the severity of the disease is related to the mutation and residual dystrophin activity [98,103,104,105]. These phenomena can only speed up the development of fibrosis after further activation of the renin–angiotensin–aldosterone system and TGF-β pathways, and this represents the molecular basis for administration of angiotensin-converting enzyme inhibitors (ACEIs), angiotensin II receptor blockers (ABs), and mineralocorticoid receptor antagonists (MRAs) as cardioprotective agents [105,106]. Overall, this evidence suggests that maintaining only DGC stability is not effective but should be integrated with therapeutic strategies that target the mechanisms governing intracellular calcium homeostasis, dysfunction of mitochondria, oxidative stress and production of endogenous NO [98,103,104].

Additionally, inherited mutations that affect the processing of the glycosylation of α-dystroglycan also affect heart development. Mutations in any of the sarcoglycans will destabilize DGC, resulting in increased fibrosis and ventricular dysfunction [107]. Though many genes have been implicated (e.g., FKTN, FKRP, ISPD, POMT1/2, POMGNT1, TMEM5, GMPPB, DPM, and LARGE), how specific genes in the pathway lead to mild to severe cardiomyopathy symptoms is still unclear. Glycosylation restorative therapeutic approaches (i.e., LARGE overexpression, ribitol/CDP-ribitol supplementation, donor sugar modulation) were successful in vivo; however, whether their long-term cardiovascular benefits are real and whether there is risk are unknown. It is still unknown whether glycosylation therapeutics could be combined with other cardioprotective methods, such as renin–angiotensin–aldosterone system (RAAS) blockade, mitochondria-directed therapies, antioxidants and antifibrotic agents, which will be beneficial.

## 8. Dystroglycan in Acquired Heart Failure

Deficient expression of dystrophin has been found in ischemic cardiomyopathy, viral myocarditis, and anthracycline-induced cardiotoxicity, indicating that compromising DGC integrity is also a player in myocardial remodeling beyond inherited muscular dystrophies [11,13,15,108,109]. Since HF is a syndrome composed of many different etiologies with varied mechanisms, prognoses and treatment strategies, the distinction between ischemic cardiomyopathy (ICM) and nonischemic cardiomyopathy (NICM) is therefore of great importance [110].

However, thus far, there is no mechanistic evidence that dystroglycan is causally involved in ischemic HF, and the involvement remains hypothetical. Among various NICM subtypes, dystroglycan seems more likely to be functionally relevant. Therefore, the loss of the DGC could be associated with changes in the ventricle through an increase in remodeling, fibrosis, and loss of contractile force in DCM patients. However, outside the context of muscular dystrophy, most patients with sporadic dilated cardiomyopathy have mutations in the Titin (TTN), Lamin A/C (LMNA), and BCL2-associated athanogene 3 (BAG3) genes, with less concrete evidence for the involvement of dystroglycan [111]. Outside the context of muscular dystrophy, the functional involvement of dystroglycan in acquired HF is currently more consistent with modifying myocardial remodeling rather than being the main driver of disease.

A newly understood pathway implicating dystroglycan in acquired HF involves the proteolytic cleavage of β-dystroglycan. A 30 kDa fragment of β-dystroglycan found in samples affected by ischemia reflected selective fragmentation, not just protein degradation [109,112]. This suggests the involvement of ischemia in β-DG proteolysis. In this process, matrix Metalloproteinase (MMP)-dependent cleavage is a plausible mechanism. Moreover, genetic deletion or pharmacological inhibition of MMP-9 leads to a rescue of β-dystroglycan, localization of neuronal nitric oxide synthase, and maintenance of membrane stability [113]. Similar cleavage of dystroglycan also occurs in other tissues, including neurons, and provides support to the concept that proteolytic degradation is a preserved process regulating cell–matrix interactions [114]. However, the specific protease(s) responsible for this degradation and their causal role in myocardial remodeling remain incompletely established.

**Figure 4:**
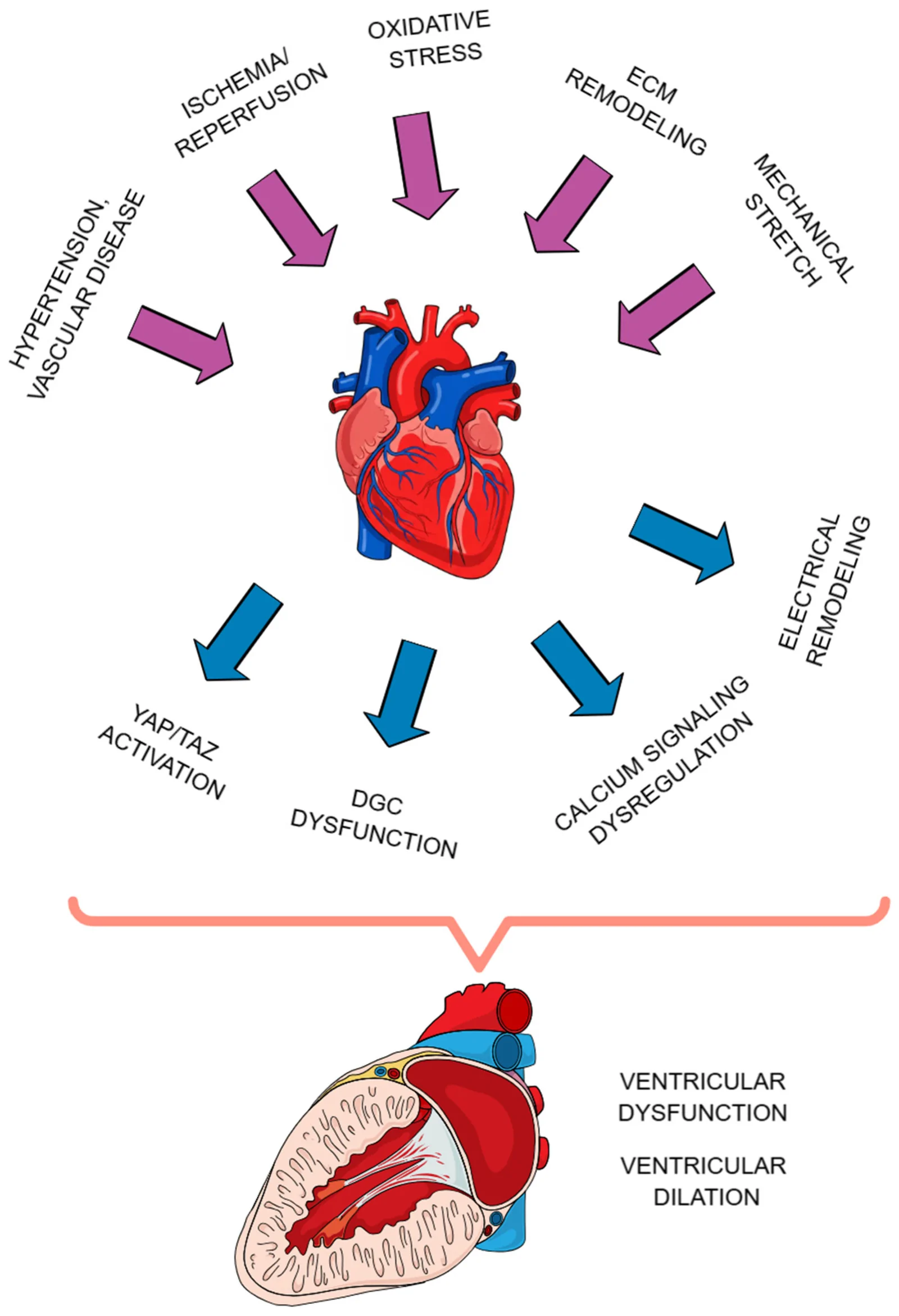
The connection between cardiac stress and DGC abnormalities associated with clinical problems. The purple arrow shows the upstream effector of cardiovascular disease and the blue arrow rep-resent the downstream molecular changes associated with cardiovascular disease.

While direct cardiac evidence is lacking, protease-mediated β-dystroglycan cleavage is thought to predispose to increased ECM–cytoskeletal uncoupling and mechanical injury during myocardial ischemic injury and fibrosis [115]. Additionally, MMP-mediated remodeling has other effects that regulate fibroblast activation, angiogenesis, and fibrosis, suggesting that the effect due to β-dystroglycan cleavage is broad [86]. Figure 4 shows the connection between cardiac stress and DGC abnormalities associated with clinical problems such as ventricular dysfunction and ventricular dilation.

## 9. Proteasome Complexes and Dystroglycan in the Heart

The disruption of dystroglycan glycosylation or DGC integrity compromises both membrane stability and regenerative capacity [17,49,116]. The ubiquitin–proteasome system (UPS) and autophagy–lysosome pathway tightly controls dystroglycan levels and maintain proteostasis in the heart. In dystroglycanopathy and muscular dystrophy models, hyperactive UPS increases DGC member turnover, which destabilizes the sarcolemma, while UPS inhibition stabilizes membrane dystroglycan/DGC and ameliorates muscle disease [34,49,117,118]. The phosphorylation induces a cascade of events that leads to DGC disassembly, and this event is required for ubiquitin-mediated degradation of β-DG and is critical for sarcolemma localization and attenuation of pathogenesis in mdx mice. It should be noted that extensive or prolonged treatment with proteasome inhibitors may adversely affect protein quality control and autophagy processes [34,117,119,120]. Future investigations will be needed to address DG proteostasis specifically in the heart and assess the therapeutic potential of modulating UPS-dependent DG turnover in cardiac remodeling, regeneration and heart failure progression.

## 10. Dystroglycan and Mitochondrial Dysfunction in the Heart

Dystroglycan helps to maintain sarcolemmal stability and the transverse tubule (T-tubule) network required for normal calcium handling. Recent studies suggest that defective α-dystroglycan glycosylation disrupts T-tubule structural integrity and increases susceptibility to cardiac damage [29]. In cardiomyocyte-specific models of dystroglycan deficiency, loss of DG causes membrane damage, progressive myocardial fibrosis, ventricular dilation, and cardiac dysfunction [46]. These findings suggest that impaired DG function is associated with cardiac injury and remodeling.

Mechanical stress in dystrophin-deficient cardiomyocytes induces excessive Ca^2+^ signaling and reactive oxygen species production, leading to mitochondrial Ca^2+^ overload and loss of mitochondrial membrane potential [121]. During the progression of dystrophic cardiomyopathy, dystrophin-deficient cardiomyocytes further develop mitochondrial oxidation, structural damage, increased mitochondrial Ca^2+^ sequestration, and membrane depolarization [122]. Moreover, impaired mitochondrial respiration is detectable before overt cardiac dysfunction in DMD model hearts [123]. On the other hand, improving mitochondrial Ca^2+^ handling in dystrophic hearts reduces mitochondrial dysfunction and cardiac fibrosis [124]. This suggests a link between mitochondrial abnormalities and cardiac remodeling.

All these suggest that DG/DGC-dependent membrane integrity and T-tubule organization may be associated with abnormal Ca^2+^ handling, mitochondrial Ca^2+^ overload and oxidative stress. This is followed by mitochondrial dysfunction, cardiomyocyte injury, and activation of cardiac remodeling. The mitochondrial mechanisms have been characterized more extensively in dystrophin-deficient hearts than in the cardiomyocyte-specific dystroglycan-deleted model.

## 11. Dystroglycan and Cardiac Fibrosis

Heart failure with preserved or reduced ejection fraction (HFpEF or HFrEF) is predominantly associated with enhanced expression of collagen types I and II and various types of other extracellular matrix (ECM) components. This elevated deposition of ECM and its subsequent accumulation result in enhanced myocardial stiffness, disturbance of electrical conduction, reduced systolic and diastolic function, and hastening the progression of failure [125,126,127]. Fibrosis, a hallmark of heart failure that contributes substantially to pathological ventricular remodeling, results in a non-contractile scar that replaces normal myocardium, and this results in progressive deterioration in ventricular performance.

Cardiovascular fibroblasts also differentiate into ECM-producing myofibroblasts through canonical and non-canonical routes. This is mediated by the transforming growth factor-β (TGF-β)/SMAD pathway, which mainly induces fibroblast activation and collagen I, collagen II, fibronectin, and α-smooth muscle actin expression. In addition, mitogen-activated protein kinase (MAPK), Phosphoinositide 3-kinase (PI3K)/Protein Kinase B (Akt), Wnt/βcatenin, and Notch-mediated noncanonical signaling amplify fibroblast activation, endothelial-to-mesenchymal transition, and matrix production [125,126,128,129]. Further ECM deposition leads to cardiac stiffness that activates mechanosensitive pathways—including integrins, focal adhesion kinase (FAK), YAP/TAZ, and transient receptor potential cation channel subfamily V member 4 (TRPV4). Interactions among these pathways create a positive feedback mechanism to maintain fibrosis. Inflammation and noncoding RNAs enhance fibrotic signaling pathways [125,130,131].

Intrinsically, dystroglycan disruption further amplifies mechanotransduction mediated by the interplay between integrins, FAK, Src, YAP/TAZ, MAPK, and PI3K/Akt, upregulating downstream TGF-β signaling and activating fibroblasts. In turn, the subsequent increased deposition of collagen reinforces the existing mechano-profibrotic signals, aggravating myocardial remodeling and causing cardiac dysfunction and arrhythmogenesis [7,47,125,126,127]. Similar evidence for this mechanism has emerged in muscular dystrophy models; in these models, the loss of the dystrophin–dystroglycan–laminin complex precedes inflammation, fibrosis, and cardiomyopathy [13,22]. Evidence for dystroglycan dysfunction leading to fibrosis in acquired human HFrEF is currently less evident.

Mitochondrial calcium overload may contribute to cardiomyocyte injury through mitochondrial membrane depolarization and activation of cell death pathways. During the progression of dystrophic cardiomyopathy, cardiomyocytes exhibit progressive mitochondrial oxidation, structural damage, increased mitochondrial Ca^2+^ sequestration, and mitochondrial membrane depolarization [122]. These abnormalities are also associated with oxidative stress and mitochondrial permeability transition in these cardiomyocytes. Furthermore, mitochondrial bioenergetic abnormalities and increased mitochondrial H_2_O_2_ emission have been detected before overt cardiac dysfunction or cardiac remodeling in dystrophic cardiomyopathy [123]. This suggests that mitochondrial dysfunction may arise at an early stage of dystrophic cardiomyopathy. Mitochondrial and metabolic remodeling can also influence the activation and persistence of cardiac myofibroblasts, suggesting a potential link between mitochondrial stress and fibrotic remodeling [132]. In addition, ROS-dependent signaling contributes directly to profibrotic fibroblast activation, as TGF-β1-induced NOX4 expression in human cardiac fibroblasts promotes ROS production, Smad2/3 phosphorylation, and α-SMA expression [133]. Therefore, these findings support the possibility that mitochondrial dysfunction may represent an intermediate linking DGC-associated disturbances in membrane integrity and calcium homeostasis to oxidative stress and fibroblast activation. This may also contribute to subsequent cardiac remodeling and fibrosis.

Restoration of dystroglycan glycosylation, strengthening of laminin–α-DG interactions, or stabilization of DGC structure is hypothesized to alleviate mechanical stress and diminish profibrotic signaling. Since fibrosis requires coordination of TGF-β, Wnt/β-catenin, YAP/TAZ and integrin–FAK signaling pathways, a combined approach targeting structural integrity and distal antifibrotic agents would likely achieve more benefits than targeting individual signaling pathways [7,125,126]. Future investigations in humans will be required to elucidate whether dystroglycan deficiency promotes fibroblast activation intrinsically or solely contributes to fibrosis secondarily to cardiomyocyte injury. Table 1 summarizes evidence for dystroglycan abnormalities associated with cardiac disease in various models, as shown below.

## 12. Therapeutic Implications

Gene therapies to restore the expression of dystrophin or to substitute for dystrophin loss have gained momentum as treatments for dystrophin-associated cardiomyopathy. Given that dystrophin loss destabilizes the dystrophin glycoprotein complex (DGC), resulting in cell membrane fragility, calcium overload, inflammation, fibrotic deposition, and heart failure, these therapies attempt to maintain the integrity of the DGC and reduce progression [142,143]. Some of the key therapeutic strategies involve CRISPR genome editing, Adeno-associated virus (AAV)-mediated microdystrophin delivery, exon-skipping antisense oligonucleotides (ASOs), RNA therapeutics, and surrogate gene therapies [144]. CRISPR/Cas9 holds the advantage of permanently correcting underlying disease-causing mutations in DMD patients. Studies on microdystrophin AAV9 delivery in a mouse model have shown promising results like restoration of cardiac dystrophin expression, decreased fibrosis, and restoration of ventricular function [145,146,147]. Moreover, translational research is challenging due to inefficient editing of postmitotic cardiomyocytes, off-target effects, immune reactions, increased vector dosages, and ambiguous long-term genetic stability [144,148].

The leading therapeutic candidate is AAV-driven replacement of microdystrophin. Microdystrophin proteins partially restore sarcolemma stability and cardiac function in preclinical studies, although they can only incompletely replicate native functions, such as nNOS localization. The method also faces challenges such as AAV vector limitations, immune response, inability to redose, and unknown long-term effects [149,150]. Exon-skipping ASOs result in partly functional dystrophin without genomic modification, and while these agents must be administered throughout life and are specific to the mutation, they remain less effective in the heart than in skeletal muscle [151,152].

Complementary approaches (that treat downstream disease mechanisms instead of dystrophin) are also useful. For example, in the case of DMD-related cardiomyopathy, the use of angiotensin-converting enzyme inhibitors [153], angiotensin receptor blockers [154] and mineralocorticoid receptor antagonists [155] has a beneficial effect. These drugs do not treat the underlying cause but prevent the symptoms. For instance, angiotensin-converting enzyme inhibitor drugs reduce cardiac afterload and mitigate fibrosis. Moreover, beta-adrenergic blockers decrease heart rate and myocardial oxygen demand, whereas mineralocorticoid receptor antagonists improve ventricular function. These drugs are also useful in the case of acquired cardiovascular disease. A review summarizing the cardioprotective effect of Sodium–glucose Cotransporter 2 inhibitors was recently reported [156].

While dystroglycan mutations have not been linked to human disease, mutations in genes including POMGnT-1, POMT1, FKTN, LARGE and FKRP can cause a broad spectrum of disease severity ranging from severe congenital muscular dystrophy, such as Walker Warburg Syndrome, to mild LGMD, such as LGMD2I [157,158,159,160]. These are the key genes regulating the glycosylation of alpha-dystroglycan, which is important for both the skeletal and cardiac muscle. Dhoke et al. show the feasibility of correcting all the mutations associated with FKRP using CRISPR-Cas9 technology in combination with patient-specific iPS cells in a mouse model [161]. However, its benefits for the heart are yet to be tested. Ragni et al. show the benefits of delivering extracellular vesicles derived from human mesenchymal stem cells containing POMT1 mRNA to myoblasts in an in vitro system, which may be useful for cardiomyocytes [162]. A review on different RNA strategies for cardiovascular disease is recommended, although it does not address dystroglycanopathies [163]. Reichart et al. tested the benefits of using an adenine base editor (ABE8e) and a potent Cas9 nuclease delivered by AAV9 to prevent hypertrophic cardiomyopathy in mice carrying the heterozygous hypertrophic cardiomyopathy pathogenic variant myosin R403Q [164]. Similarly, Chai et al. demonstrated the benefits of base editing correction for MYH7 p. R403Q and treating hypertrophic cardiomyopathy in human cardiomyocytes and humanized mice [165]. This suggests that base editing, prime editing, or RNA-targeting strategies may be useful for cardiac disease. However, their usefulness for cardiac dysfunction related to alpha dystroglycanopathy is yet to be established. Importantly, safe and efficient myocardial delivery still challenges the clinical application of the therapeutic strategy, although AAV9 exhibits better cardiac tropism among all AAVs. Limited cargo capacity, immunogenicity and inability to repeat dosing were major issues that hindered further development, leading to the generation of lipid nanoparticles and engineered nanocarriers [166,167].

Dystroglycan-targeted therapies are another potentially complementary approach. Restoring and improving the function of α-dystroglycan by introducing Large1, avoiding calpain-mediated degradation of β-dystroglycan and improving Caveolin-3 binding would potentially make the DGC stable, thereby improving membrane integrity and limiting myocardial injury [168,169,170]. These are still considered to be mainly experimental in human disease, though they would serve as an add-on therapy to dystrophin restoration by direct action on the membrane [150,168]. Overall, no single therapy fully addresses dystrophin-associated cardiomyopathy.

## 13. Future Perspectives

The involvement of dystroglycan in Hippo–YAP signaling, ECM remodeling, and the alteration of cardiomyocyte adaptation to mechanical stress is important in the context of cardiomyopathy [7,16,49,171]. It suggests dystroglycan as a key molecule that integrates structural and signaling networks for the proper functioning of the myocardium. The function of dystroglycan in acquired forms of cardiovascular diseases, however, has yet to be thoroughly evaluated. Abnormalities in dystroglycan expression, glycosylation, proteolytic cleavage and expression rate have been observed in ischemic injury, inflammatory, diabetic cardiomyopathy and chronic heart failure. However, it has not yet been fully elucidated whether these events initiate cardiac remodeling or represent a consequence of the disease state. Therefore, the current evidence indicates that dystroglycan has mainly modifying effects on cardiac remodeling and is not a primary inducer of acquired heart failure.

Nonetheless, it is unclear whether selective loss of β-DG is an event independent of dystrophin loss in acquired heart failure and whether modification of the glycosylation status of α-DG represents a general condition in ischemic and nonischemic cardiomyopathies. The mechanisms regulating dystroglycan trafficking, phosphorylation, ubiquitination, endocytosis, proteolysis, and recycling in response to cardiac stress are yet to be established. Dystroglycan has been associated with mechanotransduction, Hippo–YAP signaling, SRF signaling, calcium homeostasis, T-tubule integrity, membrane repair and ECM signaling. However, the extent to which these pathways mediate the in vivo processes of remodeling, fibrosis and contractility disorders of the ventricle is not yet defined.

The clinical translation of dystroglycan biology into treatment strategies has yet to prove successful. While attempts to rescue α-dystroglycan glycosylation by use of LARGE1, GALGT2 or ribitol supplementation have shown success in animal models of dystroglycanopathy by enhancing laminin binding and membrane integrity, their value for the treatment of acquired heart failure is still relatively uncertain. Other promising therapeutic strategies targeting β-dystroglycan stabilization, prevention of its proteolytic degradation by calpains or matrix metalloproteinases and regulation of ubiquitin–proteasome–dependent degradation are still in the early stages of development. Furthermore, the development of cardiac-specific therapeutic interventions is hampered by difficulties in cardiac delivery of the treatment. Additionally, the heterogeneity in myocardial transduction, antigen-specific responses to viral vectors and lack of validated biomarkers to evaluate dystroglycan function and its recovery also affect therapeutic interventions.

Future research needs to investigate the dynamic regulation of dystroglycan under conditions of stress instead of just protein expression levels. Combining omics data such as single-cell and spatial transcriptomics with high-resolution imaging and disease models is required to understand how dystroglycan orchestrates a broad range of cellular events. Furthermore, analysis is required to assess whether dystroglycan dysfunction/associated changes could become established as clinical indicators of heart disease progression or response to treatment. Further study is needed to establish whether the integrity of dystroglycan provides a clinical benefit across the full spectrum of heart failure presentations. Gaining insight into the in vivo functions of dystroglycan may ultimately help in understanding its role in inherited and acquired cardiomyopathies.

## Figures and Tables

**Table 1:** Dystroglycan abnormalities in cardiac disease.

Species Studied	Disease	α-DG	β-DG	Glycosylation	DGC Complex	Reference
MDX mice	DMD-associated cardiomyopathy	α-DG with altered sarcolemmal localization	Not studied	α-DG processing altered in heart	Cardiac DGC retained despite loss of dystrophin/utrophin	[134]
Aged mdx-4cv mice	Dystrophinopathy-related cardiomyopathy	Not studied	Not studied	Not studied	Reduced DGC members, including sarcoglycans and syntrophin, in dystrophic heart	[135]
Dystrophin/utrophin double knockout mice	Severe dystrophic cardiomyopathy	α-DG glycosylation changed	Not much affected	Change in glycosylation level	Cardiac DGC expressed	[134]
Largemyd mice	Glycosylation-deficient cardiomyopathy	Hypoglycosylated α-DG, reduced laminin binding	Not studied	Loss of α-DG glycosylation	Loss of DGC extracellular matrix receptor function	[50]
Cardiac myocyte DG knockout mice	Progressive dilated cardiomyopathy/heart failure	Functional absence of DG in cardiomyocytes	Functional absence of DG in cardiomyocyte	Receptor function lost	Loss of myocyte DGC is associated with fibrosis, dilation, heart failure	[46]
Cardiac fukutin-deficient mouse	Fukuyama dystroglycanopathy cardiomyopathy	Reduced α-DG glycosylation	Not studied	Glycosylation defect	Reduced sarcolemmal DGC proteins	[136]
Cardiomyopathic hamster, δ-SG mutant	HCM/DCM with sarcoglycanopathy (model animals of LGMD2F)	α-DG axis secondarily disrupted	β-DG proteolysis increased, 30 kDa fragment	Not studied	Partial disruption of DGC	[137,138]
Human	DOLK-related recessive DCM	Reduced O-mannosylation of α-DG, reduced laminin binding	Not much change as compared to control	Deficient α-DG O-mannosylation	Functional DGC impairment linked to dilated cardiomyopathy	[139]
Human	FKTN/FKRP-associated DCM	Reduced laminin binding	Not studied	Secondary glycosylation defect implied	DGC receptor dysfunction in cardiac muscle	[140]
Human	Sarcoglycan mutation	Not studied	Not studied	Not studied	Mild cardiac abnormalities	[141]

## Data Availability

No new data were created or analyzed in this study. Data sharing is not applicable to this article.

## Abbreviations

The following abbreviations are used in this manuscript:

DGC	Dystrophin–glycoprotein complex
HFrEF	Heart failure with reduced ejection fraction
Ca^2+^	Calcium
NO	Nitric oxide
DMD	Dystrophin
α-DG	α-dystroglycan
β-DG	β-dystroglycan
DRP2	Dystrophin-Related Protein 2
SG–SSPN	Sarcoglycan–sarcospan
ECM	Extracellular matrix
DCM	Dilated cardiomyopathy
FKTN	Fukutin
FKRP	Fukutin-related protein
GMPPB	GDP-mannose pyrophosphorylase B
DPM1–3	Dolichol-phosphate mannose synthase subunit 1–3
DOLK	Dolichol kinase
MPDU1	Mannose-phosphate-dolichol utilization defect 1
MG53	Mitsugumin 53
RyRs	Ryanodine receptors
SERCA	Sarcoplasmic/endoplasmic reticulum Ca^2+^-ATPase
IGF	Insulin-like growth factor
TGF-β	Transforming growth factor beta
nNOS	Nitric oxide synthases
ICM	Ischemic cardiomyopathy
NICM	Nonischemic cardiomyopathy
UPS	Ubiquitin–proteasome system
ASO	Antisense oligonucleotides
TEAD	Transcriptional enhanced associate domain
ERK	Extracellular signal-regulated kinase
BMD	Becker Muscular Dystrophy
ROS	Reactive oxygen species
ACEI	Angiotensin-converting enzyme inhibitor
ARBs	Angiotensin II receptor blockers
MRAs	Mineralocorticoid receptor antagonists
RAAS	Renin–angiotensin–aldosterone system
TTN	Titin
LMNA	Lamin A/C
BAG3	BCL2-associated athanogene 3
MMP	Matrix metalloproteinase
DG	Dystroglycan
MAPK	Mitogen-activated protein kinase
PI3K	Phosphoinositide 3-kinase
Akt	Protein Kinase B
FAK	Focal adhesion kinase
TRPV4	Transient receptor potential cation channel subfamily V member 4
AAV	Adeno-associated virus
SERCA2a	Sarcoplasmic/endoplasmic reticulum calcium ATPase 2a
SRF	Serum response factor
MRTF	Myocardin-related transcription factors
